# Equivocal PI-RADS Three Lesions on Prostate Magnetic Resonance Imaging: Risk Stratification Strategies to Avoid MRI-Targeted Biopsies

**DOI:** 10.3390/jpm10040270

**Published:** 2020-12-10

**Authors:** Daniël F. Osses, Christian Arsov, Lars Schimmöller, Ivo G. Schoots, Geert J.L.H. van Leenders, Irene Esposito, Sebastiaan Remmers, Peter Albers, Monique J. Roobol

**Affiliations:** 1 Department of Urology, Erasmus University Medical Center, 3015 GD Rotterdam, The Netherlands; s.remmers@erasmusmc.nl (S.R.); m.roobol@erasmusmc.nl (M.J.R.); 2 Department of Radiology & Nuclear Medicine, Erasmus University Medical Center, 3015 GD Rotterdam, The Netherlands; i.schoots@erasmusmc.nl; 3 Medical Faculty, Department of Urology, University Düsseldorf, D-40225 Düsseldorf, Germany; christian.arsov@med.uni-duesseldorf.de (C.A.); Peter.Albers@med.uni-duesseldorf.de (P.A.); 4 Medical Faculty, Department of Diagnostic and Interventional Radiology, University Düsseldorf, D-40225 Düsseldorf, Germany; Lars.Schimmoeller@med.uni-duesseldorf.de; 5 Department of Pathology, Erasmus University Medical Center, 3015 GD Rotterdam, The Netherlands; g.vanleenders@erasmusmc.nl; 6 Medical Faculty, Department of Pathology, University Düsseldorf, D-40225 Düsseldorf, Germany; Irene.Esposito@med.uni-duesseldorf.de

**Keywords:** clinically significant prostate cancer, equivocal PI-RADS lesions, prostate MRI, risk stratification, targeted biopsies

## Abstract

We aimed to investigate the relation between largest lesion diameter, prostate-specific antigen density (PSA-D), age, and the detection of clinically significant prostate cancer (csPCa) using first-time targeted biopsy (TBx) in men with Prostate Imaging—Reporting and Data System (PI-RADS) 3 index lesions. A total of 292 men (2013–2019) from two referral centers were included. A multivariable logistic regression analysis was performed. The discrimination and clinical utility of the built model was assessed by the area under the receiver operation curve (AUC) and decision curve analysis, respectively. A higher PSA-D and higher age were significantly related to a higher risk of detecting csPCa, while the largest index lesion diameter was not. The discrimination of the model was 0.80 (95% CI 0.73–0.87). When compared to a biopsy-all strategy, decision curve analysis showed a higher net benefit at threshold probabilities of ≥2%. Accepting a missing ≤5% of csPCa diagnoses, a risk-based approach would result in 34% of TBx sessions and 23% of low-risk PCa diagnoses being avoided. In men with PI-RADS 3 index lesions scheduled for first-time TBx, the balance between the number of TBx sessions, the detection of low-risk PCa, and the detection of csPCa does not warrant a biopsy-all strategy. To minimize the risk of missing the diagnosis of csPCa but acknowledging the need of avoiding unnecessary TBx sessions and overdiagnosis, a risk-based approach is advisable.

## 1. Introduction

The Prostate Imaging—Reporting and Data System (PI-RADS) score is important for standardized prostate magnetic resonance imaging (MRI) acquisition and reporting [1,2,3,4]. Depending on the nature of the cohort and by following the PI-RADS guidelines, a not negligible number of lesions will be scored as PI-RADS 3, which is termed equivocal [5]. The prevalence of clinically significant prostate cancer (csPCa, often defined as International Society of Urological Pathology [ISUP] grade ≥2 PCa) in biopsied PI-RADS 3 cases varies from 3% to 50% in the literature [6,7].

PI-RADS 3 lesions are challenging because their characteristics in MRI have a great overlap with benign conditions [8,9]. On the other hand, tumors that are less visible using T2-weighted (T2W) and apparent diffusion coefficient (ADC)-based tissue contrast might be classified as PI-RADS 3, despite the presence of Gleason ≥4 patterns [10]. The PI-RADS guidelines propose recommendations for MRI-directed biopsy strategies, but do not clearly state how to deal with these category 3 imaging findings. In men with an overall PI-RADS score 3 in prostate MRI, an MRI-directed biopsy should be considered; however, biopsy can be avoided or deferred in carefully chosen patients if they are not at high risk of csPCa [2]. Thus, while a targeted biopsy (TBx) may appear to be the logical next step in PI-RADS 3 cases, monitoring lesion characteristics with follow-up MRI and thereby postponing biopsies also seems to be an acceptable option [11].

The risk stratification of suspicious MRI lesions could help to avoid unnecessary biopsies. Studies on sub-classifying PI-RADS 3 lesions are limited and include most often small cohorts. The available data indicate that prostate-specific antigen density (PSA-D) might be useful in predicting the presence of csPCa [7,11,12,13,14,15,16,17]. From the perspective that size matters, subcategorizations of PI-RADS 3 lesions based on size have been proposed [18,19,20,21]. However, sufficient evidence to develop clear directions on lesion characteristic-specific management of PI-RADS 3 lesions in csPCa diagnosis is still lacking, especially in men scheduled for MRI and TBx for the first time.

Using one of the largest series of men with an overall PI-RADS score of 3 on prostate MRI undergoing a first TBx session currently available, we aimed to investigate whether stratifying PI-RADS 3 lesions based on the largest (index) lesion diameter could aid in avoiding TBx sessions and low-risk PCa diagnoses without missing the diagnosis of csPCa. In addition, acknowledging earlier publications of its potential usefulness we also studied PSA-D as stratification tool and combined this information with the largest index lesion diameter and age in a multivariable prediction model.

## 2. Materials and Methods

### 2.1. Study Population

This retrospective study was approved by the institutional review boards of the University Düsseldorf Medical Faculty and Erasmus University Medical Center with a waiver of written informed consent (NL45884.078.13/A301321).

**Figure 1:**
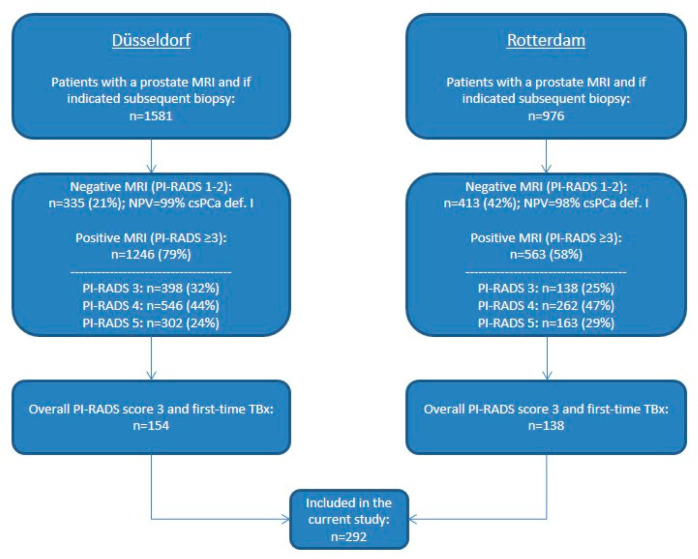
Flowchart of patients included in this study. MRI: magnetic resonance imaging; PI-RADS: Prostate Imaging Reporting and Data System; NPV: negative predictive value; csPCa: clinically significant prostate cancer; def.: definition; TBx: targeted biopsy.

Between October 2013 and July 2019, a total of 2557 consecutive men with clinical suspicion of PCa (initial/repeat biopsy) or on active surveillance (AS) for low-risk PCa underwent a multiparametric MRI (mpMRI) and, if indicated, TBx of suspicious MRI lesions with additional systematic prostate biopsy (SBx) if applicable to guidelines at one of the two tertiary referral centers. A total of 1809 men had a positive MRI, defined as an overall PI-RADS score ≥3. In the current study, we included the 292 men with an overall PI-RADS score 3 undergoing a first TBx session (Figure 1).

### 2.2. Multiparametric MRI

MRIs were performed on a 3.0-Tesla MR scanner (Magnetom Trio Siemens (Düsseldorf, Germany) or Discovery MR750 GE Healthcare (Rotterdam, The Netherlands)) with a 32-channel pelvic phased-array coil. MRI protocols included T2W, diffusion-weighted imaging (DWI) with ADC reconstructions and dynamic contrast enhanced (DCE) imaging. MRIs were reviewed by radiologists with 5 to 9 years of prostate MRI experience according to the PI-RADS version 1 or version 2 guidelines [1,4]. MRI lesions with a PI-RADS score from 3 to 5 were defined as suspicious. Prostate volume on MRI was calculated using the prolate ellipsoid formula (length × width × height × π/6). Reported largest lesion diameter measurements were indicative for TBx decision management in daily practice, not for scientific volume estimations. Largest lesion diameter was preferably measured on an axial image, or otherwise on the image which best depicted the finding. Peripheral and transition zone lesions were preferably measured on ADC and T2W imaging, respectively, or otherwise on the sequence that allowed the best visualization of the lesion.

### 2.3. Prostate Biopsy

MRI-TBx was performed using an MRI-transrectal ultrasound (TRUS) fusion system (UroNav^®^ Invivo (Düsseldorf, Germany) or UroStation^TM^ Koelis (Düsseldorf, Germany, Rotterdam, The Netherlands)). The suspicious MRI lesions were targeted with 2–5 cores per lesion, depending on the lesion size. If indicated, an additional SBx (8–12 cores, depending on the prostate volume) was performed by the same operator. All the biopsy procedures were performed by a transrectal approach and by experienced operators.

### 2.4. Pathological Review of Biopsy Specimens

Biopsy specimens were reviewed by experienced pathologists according to the ISUP 2014 modified Gleason score (GS)/Grade Group (G) system [22]. The presence of an invasive cribriform growth pattern (CR) and/or intraductal carcinoma (IDC) was recorded. In general, csPCa and thereby the recommendation for treatment was defined as any GS ≥ 3 + 4 PCa or ISUP grade ≥2 PCa found by TBx and/or SBx. Since our aim is to avoid TBx sessions, analyses are based on the TBx histopathology outcomes. The results of the SBx were not used.

### 2.5. Study Endpoints

Analyses of the largest (index) lesion diameter were performed at the patient and lesion level (= Supplementary Material). Analyses including PSA-D and age were performed at the patient level only.

Three definitions for csPCa were analyzed:

I: ISUP grade ≥2 PCa (currently the most used, = primary outcome);
II: ISUP grade ≥2 with CR and/or IDC PCa;
III: ISUP grade ≥3 PCa.

Two definitions of “TBx avoided” were used:

- patient level = a complete TBx session avoided per patient;
- lesion level = a TBx procedure avoided per lesion (= Supplementary Material).

Primary outcomes:

- PCa detection in TBx specimens of men with equivocal MRI results (PI-RADS score 3);
- results in terms of avoiding TBx sessions and the detection of low-risk PCa of a risk stratification strategy based on the largest index lesion diameter accepting missing ≤5% of csPCa diagnoses (definition I).

Secondary outcomes:

- results in terms of avoiding TBx sessions and the detection of low-risk PCa of a risk stratification strategy based on PSA-D accepting missing ≤5% of csPCa diagnoses (definition I);
- performance and clinical utility of a multivariable prediction model including age, largest index lesion diameter, and PSA-D for TBx decision management in men with equivocal MRI results (PI-RADS score 3).

### 2.6. Statistical Analysis

Descriptive statistics were used to report the patient characteristics. Categorical data are reported as count (percentage). Continuous data are reported as median (interquartile range (IQR)). Statistically significant differences in continuous non-parametric data were assessed with the Mann–Whitney U test. The Chi-square test for trend was used to test for differences in categorical data; in the case of small numbers, Fischer’s exact test was used. PSA-D was calculated by dividing the PSA level by the MRI-measured prostate volume. The relation of the largest index lesion diameter, PSA-D and age at time of biopsy, and csPCa (I) detection was assessed using (multivariable) logistic regression. Age was included as a potential predictor in order to comply with the large age range of men in daily clinical practice, and because recent prostate MRI-risk calculators have shown that age could significantly add to original PCa risk calculators [23]. For a better interpretation of the coefficients, we used the age per 10 years and multiplied the PSA-D value by 10. The discrimination of the resulting multivariable prediction model was assessed by the area under the receiver operation curve (AUC). The confidence interval of the AUC was calculated with 2000 bootstrap samples. The clinical utility of the model was assessed with decision curve analysis. Analyses were performed using Statistical Package for the Social Sciences (version 24.0; IBM, Armonk, NY, USA) and R version 3.5.1.

## 3. Results

### 3.1. Cohort Characteristics and Prostate Cancer Detection

The Düsseldorf and Rotterdam cohorts significantly differed in all reported patient characteristics, except the time of follow-up (Table 1). The majority of men had a previous negative SBx procedure, and more than one PI-RADS 3 lesion on MRI. Any PCa, csPCa (I), csPCa (II), and csPCa (III) was detected in 32% (92/292), 13% (39/292), 7% (20/292), and 3% (10/292) of all men, respectively. In men with an initial suspicion of PCa, the csPCa (I) detection was significantly lower compared to men with previous negative SBx and men on AS. In the Düsseldorf cohort consisting of mostly biopsy-naïve men (42%) and men with a previous negative biopsy (53%), csPCa (I) was detected in 3% (4/154) of men. In the Rotterdam cohort consisting of mostly men on AS (51%) and men having a previous negative biopsy (45%), csPCa (I) was detected in 25% (35/138) of men. 

The 292 men included had a total of 525 PI-RADS 3 lesions (Table 2). The median (IQR) largest lesion diameter was 11 (9–13) mm. The majority of PI-RADS 3 lesions was located in the transition zone of the prostate. In these lesions, any PCa, csPCa (I), csPCa (II), and csPCa (III) was detected in 21% (108/525), 8% (43/525), 4% (20/525), and 2% (10/525), respectively. There was no significant difference in csPCa (I) detection between peripheral- and transition-zone PI-RADS 3 lesions.

### 3.2. Risk stratification for TBx Decision Based on Largest Index Lesion Diameter

In this PI-RADS 3 cohort, in univariate analysis the largest index lesion diameter is not a statistically significant predictor (*p* = 0.70) of csPCa (I) (Table 3). It must be noted that csPCa (I) is more often detected in men with larger PI-RADS 3 index lesions compared to men with smaller index lesions. When accepting a missing ≤5% of csPCa (I) diagnoses, a threshold for largest index lesion diameter of ≥7 mm would result in 10% fewer TBx sessions and 26% of low-risk PCa diagnoses being avoided (Table 4, Supplementary Table S1). 

### 3.3. Risk Stratification for TBx Decision Based on PSA-Density

In this PI-RADS 3 cohort, in univariate analysis PSA-D is a statistically significant predictor (*p* < 0.001) of csPCa (I) (Table 3). Men with csPCa (I) had significantly higher PSA-D than men without csPCa (I). When accepting missing no more than 5% of csPCa (I) diagnoses, a PSA-D threshold of ≥0.11 ng/mL^2^ would result in avoiding 25% of TBx sessions and 11% of low-risk PCa diagnoses (Table 4).

### 3.4. Multivariable Risk Prediction for TBx Decision

**Figure 2:**
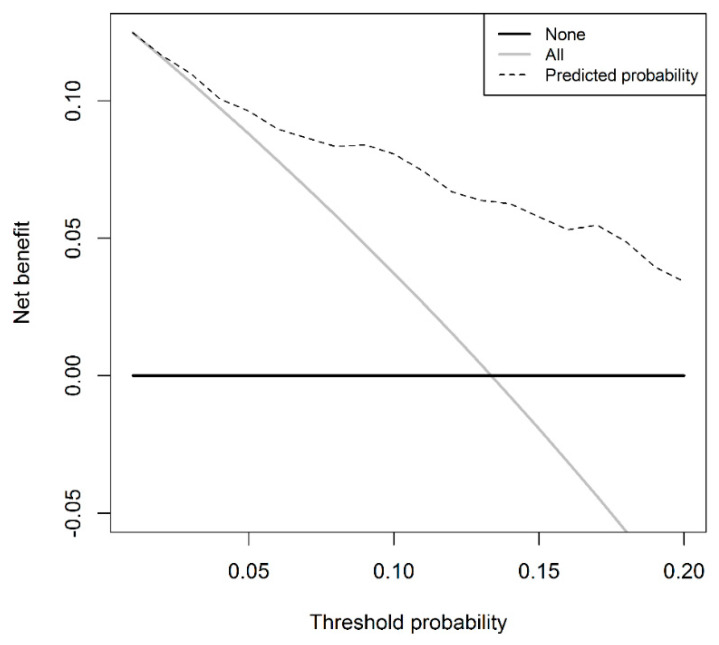
Decision curve for clinically significant prostate cancer in the targeted biopsy of the prediction model.

Multivariable logistic regression showed that a higher PSA-D and a higher age at time of biopsy were significantly (*p* ≤ 0.001) related to a higher risk of csPCa (I) diagnosis in PI-RADS 3 cases, while the largest index lesion diameter was not (*p* = 0.51) (Table 3). To avoid data-dependent variable selection for the regression model, the largest index lesion diameter is included in the model despite not being a significant predictor. The discrimination of this model is 0.80 (95% CI 0.73–0.87). The model has a higher net benefit compared to a biopsy-all PI-RADS 3 patient strategy at the threshold probability of ≥2% (Figure 2). Applying a risk-based threshold of 5% would result in 23% fewer TBx sessions and 13% fewer low-risk PCa diagnoses at the cost of missing 3% csPCa (I). Table 5 shows the numbers of avoided TBx sessions, low-risk PCa diagnoses, and missed csPCa (I) for the model using a risk threshold range of 2% to 10%. When accepting missing no more than 5% of the csPCa (I) diagnoses, a risk threshold of 7% would result in 34% fewer TBx sessions and 23% low-risk PCa diagnoses avoided. When not accepting missing the diagnosis of csPCa (I), a risk threshold of 3% should be applied, resulting in avoiding 10% of TBx sessions and almost no reduction in the diagnosis of low-risk PCa.

## 4. Discussion

Patients with PI-RADS 3 index lesions scheduled for first-time TBx represent a diagnostic problem, as there is controversy regarding whether these men should be biopsied or could safely be monitored with follow-up MRI. In this study, using a large international dataset, we found that csPCa with a detection percentage of 3–13% (based on different definitions) on first-time TBx was uncommon in PI-RADS 3 cases. This could imply that PI-RADS score 3 cases represent a category of men in whom initially monitoring with follow-up MRI could be a realistic option. The csPCa (I) detection at the patient level was 13% in our total population, with almost half of the cancers being not higher than ISUP grade 2 PCa without CR and/or IDC. Our csPCa (I) detection percentage is in line with the recent meta-analysis of Maggi et al. which included 25 studies, showing a csPCa detection percentage in PI-RADS 3 cases of 18.5% (95% CI 16.6–20.3; range 3.4–47) [7]. The reasons for the wide range of csPCa detection in PI-RADS 3 index lesions in the literature include, among others, the considerable interobserver variability in the characterization of equivocal lesions caused by reader experience and differences in technical performance, the prevalence of csPCa in different populations, and TBx-related factors. These factors could potentially also explain the difference in csPCa (I) found between the Düsseldorf and Rotterdam cohorts. The low event rate in the Düsseldorf cohort limited us to performing logistic regression analyses separately per cohort. For these analyses, the two cohorts were regarded as one, in line with analyses on heterogeneous cohorts [24].

Instead of a monitoring all or a biopsy-all PI-RADS 3 patient strategy, a more realistic approach to avoid TBx sessions and low-risk PCa diagnoses would be to apply a risk stratification strategy. Risk stratification for TBx decision solely based on the largest index lesion diameter did not aid in avoiding TBx sessions while assuring csPCa (I) detection. On the contrary, risk stratification based on PSA-D only or a multivariable approach including the next to largest index lesion diameter, PSA-D, and age could result in avoiding a substantial number of TBx sessions and low-risk PCa diagnoses at the cost of missing only limited numbers of csPCa (I) diagnoses. These results suggest that when TBx is considered in men with PI-RADS 3 index lesions scheduled for first-time TBx, risk stratification based on PSA-D or preferably a multivariable model-based risk stratification approach is advisable.

To the best of our knowledge, this is the first study to investigate largest (index) lesion diameter as a stratification tool in a large daily clinical cohort of men with PI-RADS 3 index lesions. According to our results, the largest index lesion diameter is not a significant predictor of csPCa in PI-RADS 3 cases. To lower the risk of statistical overfitting, largest index lesion diameter is included in the multivariable prediction model [25]. In our cohort, slightly more csPCa (I) was detected in PI-RADS 3 index lesions with a diameter ≥10 mm (14%, 31/217), compared to index lesions with a diameter <10 mm (11%, 8/75). This finding could suggest that csPCa is relatively rare in smaller PI-RADS 3 index lesions. Rais-Bahrami et al. suggest that small MRI index lesions (≤7 mm) may correspond to benign lesions or indolent cancers [18]. Furthermore, Rosenkrantz et al. proposed to upgrade a PI-RADS 3 to a PI-RADS 4 lesion on the basis of larger size [20,26]. These assumptions do, however, not take into account the scenario that in the studied series small PI-RADS 3 index lesions harboring csPCa could have been mis-sampled by TBx. The absence of csPCa in the TBx specimens would then mean that csPCa was missed and not that there was no csPCa present [6,27]. However, if this would really be the case follow-up of the lesions with MRI could overcome the problem of missing a timely csPCa diagnosis.

PSA-D showed to be a significant clinical predictor of csPCa in our cohort. Applying solely PSA-D as risk stratification tool could result in 25% less TBx sessions and 11% less low-risk PCa diagnoses, missing only 5% csPCa (I). This high predictive value of PSA-D in PI-RADS 3 cases is in line with previous studies, and also with studies reporting on TBx and SBx histopathology outcomes [17]. Venderink et al. showed that offering a biopsy to only PI-RADS 3 men with a PSA-D of ≥0.15 ng/mL^2^ resulted in 42% of biopsy sessions avoided at the cost of missing 6% csPCa. Lowering the threshold to ≥0.12 ng/mL^2^ would result in 26% of biopsy sessions avoided, missing no csPCa [28]. Therefore, PSA-D may represent a good index to decide which PI-RADS 3 men should undergo a biopsy [29]. The risk stratification of PI-RADS 3 cases could further be improved by a model-based approach in which PSA-D, largest index lesion diameter, and age are combined in a multivariable prediction model that predicts the risk of csPCa of a PI-RADS 3 man, as shown by our findings. To the best of our knowledge, our study is one of the first studies, next to the work of Di Trapani et al., to show the high added value of such a model-based approach in safely avoiding TBx sessions and low-risk PCa diagnoses in specifically PI-RADS 3 cases [29].

Next to the most often used definitions for csPCa, we studied the prevalence of PI-RADS 3 lesions related to the presence of CR and IDC in TBx specimens. CR and IDC are prognostic drivers in cancer-specific survival, even more than other Gleason 4 patterns [30,31]. Although ISUP grade ≥2 PCa was our primary outcome measure for csPCa, the incorporation of this secondary growth pattern information into the risk stratification could further improve the selection of men who will benefit from treatment, especially because almost half of the detected ISUP grade ≥2 PCa in our cohort was ISUP grade 2 without CR and/or IDC PCa. Therefore, we may argue that the threshold for csPCa should be ISUP grade ≥2 with CR and/or IDC PCa to save even more TBx sessions in men with PI-RADS 3 index lesions and thereby avoid the (over)detection of ISUP grade 2 PCa, which potentially could never harm a patient if left undetected [32].

The strength of our study is the inclusion of data from two centers, resulting in one of the largest series of men with an overall PI-RADS score 3 undergoing first-time TBx. This makes our study results more generally representative by giving more an overall view of the real-world setting of PI-RADS 3 lesions in daily practice, compared to reporting single center results. It must, however, be noted that every institution should know their own test performance statistics when making clinical decisions based on prostate MRI findings, because of existing differences in radiology, fusion biopsy and pathology learning curves per institution [33]. Furthermore, our analysis of different csPCa definitions including the presence of secondary growth patterns is of high added value for further clinical decision-making.

Some limitations of our study should be highlighted. First, our study has a retrospective design and could thereby introduce a selection bias. However, our study represents a cohort of consecutive men. Second, men included were treated over a long time frame in which changes in the PI-RADS classification also occurred. However, the newer PI-RADS versions may not necessarily be better regarding diagnostic accuracy than the original PI-RADS version [34,35,36]. Third, we did not include SBx or prostatectomy outcomes as a reference standard in our analyses. Some literature suggests performing a combined biopsy strategy in PI-RADS 3 cases [7]. However, since our primary objective was to establish directions for the lesion characteristic-specific management of PI-RADS 3 lesions in csPCa diagnosis, SBx outcomes would not have been of added value in answering our research questions. Furthermore, our results are similar to studies investigating PSA-D as a stratification tool but reporting on TBx and SBx outcomes [17]. This suggests that the TBx-only strategy could be similar in csPCa detection to the combined biopsy strategy in PI-RADS 3 cases. Nevertheless, when considering a biopsy in PI-RADS 3 men, we advise that adding SBx to TBx should be discussed at an individual level taking into account the benefits and harms. Fourth, although we have found potential predictors of csPCa in men with a PI-RADS 3 index lesion, the constructed prediction model is not (yet) usable in clinical practice for TBx decision management. To construct a more robust prediction model for TBx decision in PI-RADS 3 cases, more data are necessary and an external validation of the model is advised before its application in clinical practice. Lastly, the lesion measurements, although measured according to the PI-RADS recommendations, were not standardized. We acknowledge that standardized MRI lesion measurement should be the gold standard [37,38]. However, as long as this is not implemented in routine clinical practice, lesion measurement according to the PI-RADS guidelines is the daily workflow in most hospitals.

## 5. Conclusions

Overall, in men with PI-RADS 3 index lesions scheduled for first-time TBx, the balance between the number of TBx sessions, the detection of low-risk PCa, and the detection of csPCa does not warrant a biopsy-all strategy. If the (low) risk of not diagnosing csPCa in these men is accepted, monitoring these men with follow-up prostate MRI could be considered as the optimal strategy avoiding TBx and the detection of low-risk PCa. To minimize the risk of missing the diagnosis of csPCa while acknowledging the need to avoid unnecessary TBx sessions and overdiagnosis, a model-based risk stratification approach including at least PSA-D could be considered. More data are necessary to construct a robust clinically useful prediction model for TBx decision management in PI-RADS 3 cases. Future large-scale studies should also focus on the need, optimal intervals, and frequencies for follow-up MRIs in these men.

## Figures and Tables

**Table 1:** Patient characteristics.

	Total Cohort (*n* = 292)	Düsseldorf (*n* = 154)	Rotterdam (*n* = 138)	*p Value*
**Age (yr), median (IQR)**	64 (58–69)	61 (53–67)	67 (61–72)	*<0.001*
**Follow-up time (yr), median (IQR)**	2 (1–3)	3 (2–4)	2 (1–3)	*0.083*
**PSA level (ng/mL), median (IQR)**	8.1 (6–12.2)	7.3 (5.5–11)	9.5 (6.6–13.1)	*0.001*
**Prostate volume on MRI (mL), median (IQR)**	52.3 (36–78.8)	58 (44.8–83.3)	46 (31–68.5)	*<0.001*
**PSA-density (ng/mL/mL), median (IQR)**	0.16 (0.11–0.25)	0.13 (0.1–0.18)	0.23 (0.13–0.31)	*<0.001*
**Indication of prostate MRI, no. (%)**				
**Initial PCa diagnosis**	69 (24)	64 (42)	5 (4)	*<0.001*
**Previous negative biopsy**	144 (49)	82 (53)	62 (45)	
**Active Surveillance**	79 (27)	8 (5)	71 (51)	
**DRE findings, no. (%)**				
**Benign**	191 (65)	75 (49)	116 (84)	*<0.001*
**Suspected**	60 (21)	38 (25)	22 (16)	
**Unknown**	41 (14)	41 (27)	0 (0)	
**PI-RADS 3 lesions on MRI, no. (%)**				
**1**	132 (45)	16 (10)	116 (84)	*<0.001*
**2**	89 (31)	69 (45)	20 (15)	
**3**	69 (24)	67 (44)	2 (1)	
**4**	2 (1)	2 (1)	0 (0)	
**Highest grade at TBx, no. (%)**				
**no PCa**	200 (69)	141 (92)	59 (43)	*<0.001*
**G 1**	53 (18)	9 (6)	44 (32)	
**G 2**	19 (7)	2 (1)	17 (12)	
**G 2 with CR and/or IDC**	10 (3)	0 (0)	10 (7)	
**G 3**	9 (3)	2 (1)	7 (5)	
**G 4–5**	1 (1)	0 (0)	1 (1)	

IQR: interquartile range; PSA: prostate-specific antigen; MRI: magnetic resonance imaging; PCa: prostate cancer; DRE: digital rectal examination; PI-RADS: Prostate Imaging Reporting and Data System; TBx: targeted biopsy; G: grade; CR: cribriform growth pattern; IDC: intraductal carcinoma.

**Table 2:** PI-RADS 3 lesion characteristics.

	Total PI-RADS 3 Lesions (*n* = 525)	PI-RADS 3 Lesions Düsseldorf (*n* = 363)	PI-RADS 3 Lesions Rotterdam (*n* = 162)	*p Value*
**Lesion size (largest diameter, mm), median (IQR)**	11 (9–13)	12 (10–13)	10 (7–12)	*<0.001*
**Lesion zone, no. (%)**				
**Peripheral zone**	186 (35)	107 (30)	79 (49)	*<0.001*
**Transition zone**	324 (62)	246 (68)	78 (48)	
**Central zone**	2 (1)	0 (0)	2 (1)	
**>1 zone**	13 (3)	10 (3)	3 (2)	
**Lesion location, no. (%)**				
**Anterior**	340 (65)	258 (71)	82 (51)	*<0.001*
**Posterior**	178 (34)	103 (28)	75 (46)	
**Both**	7 (1)	2 (1)	5 (3)	
**Grade at TBx, no. (%)**				
**no PCa**	417 (79)	346 (95)	71 (44)	*<0.001*
**G 1**	65 (12)	10 (3)	55 (34)	
**G 2**	23 (4)	5 (1)	18 (11)	
**G 2 with CR and/or IDC**	10 (2)	0 (0)	10 (6)	
**G 3**	9 (2)	2 (1)	7 (4)	
**G 4–5**	1 (1)	0 (0)	1 (1)	

IQR: interquartile range; TBx: targeted biopsy; PCa: prostate cancer; G: grade; CR: cribriform growth pattern; IDC: intraductal carcinoma; PI-RADS: Prostate Imaging Reporting and Data System.

**Table 3:** Output of the logistic regression analyses.

	Univariable Analysis			Multivariable Analysis (Model)		
Variable	Odds Ratio	95% CI	*p Value*	Odds Ratio	95% CI	*p Value*
**Age (per 10 years)**	2.62	1.62–4.25	*<0.001*	2.31	1.43–3.92	*0.001*
**Largest index lesion diameter**	1.01	0.96–1.01	*0.70*	1.02	0.96–1.08	*0.51*
**PSA-density (multiplied by 10)**	1.65	1.31–2.10	*<0.001*	1.53	1.21–1.97	*<0.001*

PSA: prostate-specific antigen.

**Table 4:** Summary table of the avoided targeted biopsy sessions and prostate cancer diagnoses missed when using the largest index lesion diameter or PSA density as a stratification tool.

Thresholds		TBx Sessions	ISUP Grade 1 PCa	ISUP Grade ≥2 PCa	ISUP grade ≥2 with CR and/or IDC PCa	ISUP Grade ≥3 PCa
Largest Index Lesion Diameter	PSA-Density	Avoided (*n*, %)	Not Detected (*n*, %)	Missed Diagnosis (*n*, %)	Missed Diagnosis (*n*, %)	Missed Diagnosis (*n*, %)
**Monitor all patients**		292 (100%)	53 (100%)	39 (100%)	20 (100%)	10 (100%)
**Biopsy all patients**		0 (%)	0 (0%)	0 (0%)	0 (0%)	0 (0%)
**≥4 mm**	-	3 (1%)	3 (6%)	0 (0%)	0 (0%)	0 (0%)
**≥5 mm**	-	11 (4%)	5 (9%)	1 (3%)	1 (5%)	0 (0%)
**≥6 mm**	-	19 (7%)	10 (19%)	1 (3%)	1 (5%)	0 (0%)
**≥7 mm**	-	29 (10%)	14 (26%)	2 (5%)	2 (10%)	1 (10%)
**≥8 mm**	-	41 (14%)	16 (30%)	5 (13%)	3 (15%)	2 (20%)
**-**	**≥0.05 ng/mL^2^**	5 (2%)	0 (0%)	0 (0%)	0 (0%)	0 (0%)
**-**	**≥0.10 ng/mL^2^**	55 (20%)	4 (1%)	2 (5%)	1 (5%)	1 (10%)
**-**	**≥0.11 ng/mL^2^**	73 (25%)	6 (11%)	2 (5%)	1 (5%)	1 (10%)
**-**	**≥0.12 ng/mL^2^**	91 (31%)	9 (17%)	3 (8%)	1 (5%)	1 (10%)
**-**	**≥0.15 ng/mL^2^**	141 (48%)	14 (26%)	5 (13%)	1 (5%)	1 (10%)
**-**	**≥0.20 ng/mL^2^**	183 (63%)	19 (36%)	10 (26%)	2 (10%)	2 (20%)

PSA: prostate-specific antigen; TBx: targeted biopsy; ISUP: International Society of Urological Pathology; PCa: prostate cancer; CR: cribriform growth pattern; IDC: intraductal carcinoma.

**Table 5:** Performance and clinical utility of the prediction model: numbers of avoided TBx sessions, low-risk PCa diagnoses, and missed csPCa using a risk threshold range of 2% to 10%.

Threshold	TBx Sessions	ISUP Grade 1 PCa	ISUP Grade ≥2 PCa
Risk of csPCa	Avoided (*n*, %)	Not Detected (*n*, %)	Missed Diagnosis (*n*, %)
**Monitor all patients**	292 (100%)	53 (100%)	39 (100%)
**Biopsy all patients**	0 (0%)	0 (0%)	0 (0%)
**≥2%**	10 (3%)	1 (2%)	0 (0%)
**≥3%**	30 (10%)	2 (4%)	0 (0%)
**≥4%**	47 (16%)	4 (8%)	1 (3%)
**≥5%**	65 (23%)	7 (13%)	1 (3%)
**≥6%**	84 (30%)	9 (17%)	2 (5%)
**≥7%**	97 (34%)	12 (23%)	2 (5%)
**≥8%**	119 (42%)	16 (30%)	3 (8%)
**≥9%**	137 (48%)	20 (38%)	3 (8%)
**≥10%**	150 (53%)	22 (42%)	4 (10%)

CsPCa: clinically significant prostate cancer; TBx: targeted biopsy; ISUP: International Society of Urological Pathology; PCa: prostate cancer.

## Supplementary Materials

The following are available online at https://www.mdpi.com/2075-4426/10/4/270/s1: Table S1: Performed and avoided targeted biopsy sessions and numbers of prostate cancer diagnoses detected and missed in PI-RADS 3 patients with different thresholds for clinically significant prostate cancer, when using largest index lesion diameter only as stratification tool. TBx: targeted biopsy; ISUP: International Society of Urological Pathology; PCa: prostate cancer; CR: cribriform growth pattern; IDC: intraductal carcinoma. Table S2: Performed and avoided targeted biopsies procedures and numbers of prostate cancer diagnoses detected and missed in PI-RADS 3 lesions with different thresholds for clinically significant prostate cancer, when using largest lesion diameter as stratification tool. TBx: targeted biopsy; ISUP: International Society of Urological Pathology; PCa: prostate cancer; CR: cribriform growth pattern; IDC: intraductal carcinoma. Table S3: Specifications of applied mpMRI scan protocols. TSE: turbo spin echo; EPI: echo-planar imaging; DWI: diffusion weighted imaging; DCE: dynamic contrast enhanced; TR: repetition time; TE: echo time; FOV: field of view; NEX: number of excitation; H: head; F: feet; R: right; L: left.

## Abbreviations

ADC	apparent diffusion coefficient
AUC	area under the receiver operation curve
AS	active surveillance
CR	cribriform growth pattern
csPCa	clinically significant prostate cancer
DCE	dynamic contrast enhanced
DRE	digital rectal examination
DWI	diffusion weighted imaging
G	ISUP grade
GS	Gleason score
IDC	intraductal carcinoma
IQR	interquartile range
ISUP	International Society of Urological Pathology
mpMRI	multiparametric MRI
MRI	magnetic resonance imaging
MRI ± TBx	MRI with or without TBx
PCa	prostate cancer
PI-RADS	Prostate Imaging—Reporting and Data System
PSA	prostate-specific antigen
PSA-D	PSA-density
SBx	systematic prostate biopsy
T2W	T2-weighted
TBx	targeted biopsy
TRUS	transrectal ultrasound
