# Supplementary material for: Equivocal PI-RADS Three Lesions on Prostate Magnetic Resonance Imaging: Risk Stratification Strategies to Avoid MRI-Targeted Biopsies
Source: J Pers Med. 2020 Dec 10;10(4):270. doi: 10.3390/jpm10040270 (PMC7768373; doi:10.3390/jpm10040270)
Supplement: Supplementary file 1 [file jpm-10-00270-s001.zip › Suppl. Table 3._FINAL.docx]

| **Discovery MR750**  **GE Healthcare** | **Sequence** | **T2 TSE** | **T2 TSE** | **T2 TSE** | **EPI DWI** | **T1 LAVA/FLEX** | **DCE dynamic**  **T1 LAVA** |
| --- | --- | --- | --- | --- | --- | --- | --- |
|  | **Orientation** | Sagittal | Coronal | Axial | Axial | Axial | Axial |
|  | **TR (ms)** | 8941 | n.a. | 5660 | 3203 | - | - |
|  | **TE (ms)** | 140 | n.a. | 140 | Min. | - | - |
|  | **Flip Angle (deg)** | 125 | n.a. | 125 | - | 12 | 12 |
|  | **Matrix size** | 256 | n.a. | 320 | 128 | 300 | 160 |
|  | **# Slices/ Thickness(mm)** | 30 slices  3 mm | n.a. | 30 slices  3 mm | 30 slices  3 mm | 24 slices  4 mm | 24 slices  4 mm |
|  | **Gap** | 0% | n.a. | 0% | 0 % | - | - |
|  | **Voxel size (mm)** | 0.7x0.7x3 | n.a. | 0.6x0.6x3 | 2x2x3 | 1.2x1.2x4 | 1.5x1.5x3 |
|  | **Averages/NEX** | 2 | n.a. | 4 | - | - | - |
|  | **FOV** | 190 | n.a. | 190 | 240 | 360 | 240 |
|  | **Phase enc Dir** | S/I | n.a. | A/P | Unswap | R/L | R/L |
|  | **Fat suppres** | None | n.a. | None | Fat sat. | None | None |
|  | **b-values (sec/mm2)** | - | n.a. | - | 50, 400, 800, 1500 (calculated) | - | - |
|  | **Measurements** | 1 | n.a. | 1 | 1 | 1 | 20 |
|  | **Contrast agent** | - | n.a. | - | - | - | 0.1 mmol/Kg gadolinium |
|  | **Acquisition time** | 2:58 | n.a. | 4:39 | 5:04 | 3:07 | 3:02 |
| **Magnetom Trio® Siemens** | **Sequence** | **T2 TSE** | **T2 TSE** | **T2 TSE** | **EPI DWI** | **T1 TSE** | **DCE dynamic**  **T1 Vibe** |
|  | **Orientation** | Sagittal | Coronal | Axial | Axial | Axial | Axial |
|  | **TR (ms)** | 11330 | 11330 | 10630 | 4700 | 650 | 3.62 |
|  | **TE (ms)** | 103 | 103 | 117 | 90 | 13 | 1.27 |
|  | **Flip Angle (deg)** | 150 | 150 | 150 | - | - | 12 |
|  | **Matrix size** | 256 | 256 | 256 | 136 | 320 | 128 |
|  | **# Slices/ Thickness(mm)** | 30 slices  3 mm | 30 slices  3 mm | 30 slices  3 mm | 30 slices  3 mm | 30 slices  3 mm | 20 slices  3 mm |
|  | **Gap** | 0% | 0% | 0% | 0 % | - | - |
|  | **Voxel size (mm)** | 0.7x0.7x3 | 0.7x0.7x3 | 0.5x0.5x3 | 1.5x1.5x3 | 1.3x0.9x5 | 1.5x1.5x3.3 |
|  | **Averages/NEX** | 1 | 1 | 2 | 8 | - | - |
|  | **FOV** | 170 | 170 | 128 | 200 | 300 | 192 |
|  | **Phase enc Dir** | H>>F | R>>L | R>>L | R>>L | R>>L | R>>L |
|  | **Fat suppres** | None | None | None | Fat sat. | None | None |
|  | **b-values (sec/mm2)** | - | - | - | 0, 500,  1000  + 1400 (acquired) | - | - |
|  | **Measurements** | 1 | 1 | 1 | 1 | 1 | 22 |
|  | **Contrast agent** | - | - | - | - |  | 0.1 mmol/Kg gadolinium |
|  | **Acquisition time** | 4:11 | 4:11 | 8:21 | 4:31 | 5:15 | 3:36 |
